# Comparative Analyses of Lipoprotein Lipase, Hepatic Lipase, and Endothelial Lipase, and Their Binding Properties with Known Inhibitors

**DOI:** 10.1371/journal.pone.0072146

**Published:** 2013-08-21

**Authors:** Ziyun Wang, Shen Li, Lidan Sun, Jianglin Fan, Zhenming Liu

**Affiliations:** 1 State Key Laboratory of Natural and Biomimetic Drugs, School of Pharmaceutical Sciences, Peking University, Beijing, P. R. China; 2 Department of Molecular Pathology, Interdisciplinary Graduate School of Medicine and Engineering, University of Yamanashi, Yamanashi, Japan; University of Edinburgh, United Kingdom

## Abstract

The triglyceride lipase gene subfamily plays a central role in lipid and lipoprotein metabolism. There are three members of this subfamily: lipoprotein lipase, hepatic lipase, and endothelial lipase. Although these lipases are implicated in the pathophysiology of hyperlipidemia and atherosclerosis, their structures have not been fully solved. In the current study, we established homology models of these three lipases, and carried out analysis of their activity sites. In addition, we investigated the kinetic characteristics for the catalytic residues using a molecular dynamics simulation strategy. To elucidate the molecular interactions and determine potential key residues involved in the binding to lipase inhibitors, we analyzed the binding pockets and binding poses of known inhibitors of the three lipases. We identified the spatial consensus catalytic triad “Ser-Asp-His”, a characteristic motif in all three lipases. Furthermore, we found that the spatial characteristics of the binding pockets of the lipase molecules play a key role in ligand recognition, binding poses, and affinities. To the best of our knowledge, this is the first report that systematically builds homology models of all the triglyceride lipase gene subfamily members. Our data provide novel insights into the molecular structures of lipases and their structure-function relationship, and thus provides groundwork for functional probe design towards lipase-based therapeutic inhibitors for the treatment of hyperlipidemia and atherosclerosis.

## Introduction

The triglyceride lipase gene subfamily (TLGS) is comprised of three evolutionarily related lipases: lipoprotein lipase (LPL), hepatic lipase (HL), and endothelial lipase (EL), and plays a central role in plasma lipoprotein metabolism and homeostasis [1]. These lipases are differentiated by their tissue-specific gene expression, and substrate specificity. LPL is mainly expressed in adipose and muscle tissues, while HL is specifically expressed in the liver [2], [3]. In contrast, EL is a newly identified lipase that is synthesized by vascular endothelial cells, thyroid epithelial cells, and hepatocytes [4]. LPL mainly hydrolyzes the triglycerides of chylomicrons and very low-density lipoproteins, whereas EL exerts significant phospholipase activity on high-density lipoprotein (HDL) particles, but has less triglyceride lipase activity [2], [4]–[6]. HL seems to have equal hydrolytic activity on triglycerides, phospholipids of remnant lipoproteins, and HDL particles [7]. Furthermore, all lipases are expressed in macrophages and have been implicated in the pathogenesis of atherosclerosis [7]–[10].

Because of their diverse range of important functions in maintaining lipoprotein homeostasis and their involvement in the pathophysiology of hyperlipidemia and atherosclerosis, the TLGS members are attractive biomarkers and potential therapeutic targets for the treatment of metabolic diseases [11]. For example, the up-regulation of LPL activity may be beneficial in obesity and diabetes, whereas inhibition of EL may increase plasma HDL levels [12], [13]. It is therefore essential to obtain molecular structural information to elucidate how these lipases exert their effects, and how they interact with their ligands.

Previous studies have revealed that these lipases share common motifs, including a heparin-binding domain, and key active site residues (called the α/β hydrolase fold) [14]. The active site residues are responsible for maintaining the juxtaposition of the conserved residues in the active site pentapeptide, and evolved independently from the forces that constrained and molded the analogous pentapeptide of serine proteases [15]. It is likely that these two motifs are a result of convergent evolution [16]. Each lipase molecule has a lid element, which blocks the enzymatic active site, and cofactors that are required for enzymatic activation. For example, apolipoprotein C-II (apoC-II) is a cofactor for LPL activation, while the cofactors for HL and EL are still not fully defined [17]. Site-directed mutagenesis studies showed that LPL and HL, along with pancreatic lipase (PL), contain a serine residue within the GXSXG sequence as an acylated center [18]–[20]. Previous studies also revealed that LPL and HL belong to the group of two-domain enzymes [21], [22]. However, in spite of the progress in understanding the functions of lipases, information on how the ligands interact with each lipase has not been reported due to the lack of X-ray crystallographic structures. This may hinder a precise understanding of their physiological functions, pathophysiological significance, and the design of effective inhibitors for clinical applications.

In this study, we used a computational strategy including homology modeling, molecular dynamics simulation (MDS), binding site detection and docking validation. The aims of this strategy were: (1) Homology modeling and comparison of the structures of LPL, HL and EL. This is the first attempt to generate the 3-dimensional (3D) homology modelled structures of all the TLGS members simultaneously. Since they belong to the same subfamily, the comparison might be expected to explain the differences of their functions stemming from structural differences; (2) The motion of the catalytic triad and key residues within the binding pockets, which will provide important information on the substrate catalytic process; (3) The binding poses of known inhibitors, especially specific and non-specific inhibitors, to compare the binding characteristics; and (4) Modeling of comprehensive 3D models for these lipases, which can be used for further drug design applications such as virtual screening and detailed protein-ligand reciprocity.

## Materials and Methods

### Sequence Analyses of LPL, HL, and EL

The human sequences of LPL, HL, and EL were acquired from the PubMed database (accession numbers EAW63764, AAA59521, and AAD30434; http://www.ncbi.nlm.nih.gov/entrez). Discovery Studio 2.5 (DS 2.5; Accelrys, Inc.) was used for all the sequence studies. Sequence similarity searching was then carried out using BLAST searches against the protein data bank [23] to find crystal structures serving as templates for the homology modeling. Alignments between the template sequences and the sequences of TLGS members (target sequences) were then performed, which were used for further homology modeling. Finally, site-directed mutagenesis studies showed that LPL and HL, along with PL, contain a serine residue within the GXSXG sequence as an acylated center. We therefore carried out multiple-alignment of TLGS members against PL, and used this information for initial identification of the typical “Ser-Asp-His” characteristics of TLGS members [24].

### Homology Modeling of LPL, HL and EL

Homology modeling was done using the templates identified above, and DS 2.5 was used to generate the models of TLGS members. Modeller9v4 auto-modeling strategy was then used to build ten homology models, without hydrogen atoms, for each TLGS member. Accordingly, thirty models were built by optimization of the molecular probability density function, which uses a variable target function procedure in Cartesian space that employs methods of conjugate gradients and molecular dynamics with simulated annealing. The model that has the lowest molecular probability density function score was selected from each group, and the root mean square deviation (RMSD) value was calculated for further computational study.

Through the procedure mentioned above, three initial models were constructed, before being validated by PROCHECK [25], the profile-3D module of DS 2.5 (see Table 1), and ProSA analysis (https://prosa.services.came.sbg.ac.at/prosa.php) (see Table 2). The profile-3D method measures the compatibility of an amino acid sequence with a known 3D protein structure, and ProSA evaluates the energy of the structure using distance pair potential. Residues with negative ProSA scores confirm the reliability of the model.

**Table 1:** Validation of models before and after MDS by profile-3D.

	Verify Score	Verify Expected High Score	Verify Expected Low Score
**LPL Before MDS**	161.83	199.91	89.96
**LPL After MDS**	185.58	199.91	89.96
**HL Before MDS**	182.87	212.34	95.55
**HL After MDS**	187.57	212.34	95.55
**EL Before MDS**	162.05	212.34	95.55
**EL After MDS**	184.18	212.34	95.55

**Table 2:** Validation of models before and after MDS by ProSA analysis.

ProSA Score	Before MDS	After MDS
**LPL**	−6.65	−7.37
**HL**	−6.95	−7.20
**EL**	−6.65	−7.37

### MDS

MDS was carried out to examine the quality of the homology models by assessing their stability. Three models were placed in respective 1.0 nm cubic boxes, and refined with the GROMACS package using the GROMOS96 force field [26]. The simple point charge water model was used to create the aqueous environment [27], [28]. Periodic boundary conditions were applied, and the systems were neutralized using the appropriate counter ions (Cl^−^). To reduce the effect of unfavorable interactions produced by solvents and ion generation, each system was subjected to 5000 steps of energy minimization using conjugate gradient methods [29]. The models were further subjected to full MDS with Particle Mesh Ewald ensembles for a period of 4000 ps without restraints [30], and the Berendsen coupling scheme was used with ensembles [26]. The LINCS algorithm was used to constrain all bond lengths [31], while the SETTLE algorithm was used to constrain the geometry of water molecules [32]. Following these methods, the quality of the initial models was improved. After the optimization procedure, three refined models were obtained and further assessed using profile-3D in DS 2.5 (Table 1), and ProSA analysis (Table 2).

### Detection and Selection of Binding Pockets

The binding pockets of the lipase models were derived from MDS results, and further studied by Cavity in LigBuilder V2.0 Program [33], [34]. Lai et al at Peking University recently developed Cavity, for identifying protein-binding sites and characterizing druggable ligand-binding pockets. It was used to estimate the potential best binding affinity of each proposed binding pocket. A score, as a function of geometric shape, hydrogen bonding, and hydrophobic effect for each cavity, was calculated. The ultimate ligand-binding pocket of each lipase was selected in reference to the spatial location of the catalytic triad.

### Docking Analyses

AutoDock Vina was used to carry out docking analysis [35], [36]. The lipase models constructed were used as the receptors for docking. Binding pockets were used as the center of the grid boxes for docking, and the size of each box was assigned as 20×20×20 Å. To account for side chain flexibility during docking, flexible torsions in the ligands were assigned using AutoDockTools, and the acyclic dihedral angles were allowed to rotate freely. Docking calculations were performed using the default procedure implemented in AutoDock Vina, and the binding pose with the lowest binding energy was selected as representative to show the binding mode of the ligands.

The molecular docking study included two tasks. Firstly, known inhibitors of TLGS members, extracted from the ChEMBL database [37], were docked to determine if the proposed binding pockets were suitable for their binding. Meanwhile, consistency between the virtual computed results, and biological experiment results, was used to judge whether the obtained models could be used reliably for protein-ligand interaction studies or virtual screening. Secondly, specific and non-specific inhibitors for LPL, HL, and EL were studied in detail to characterize the similarities and differences of their binding poses.

### Ligand Dataset

**Figure 1:**
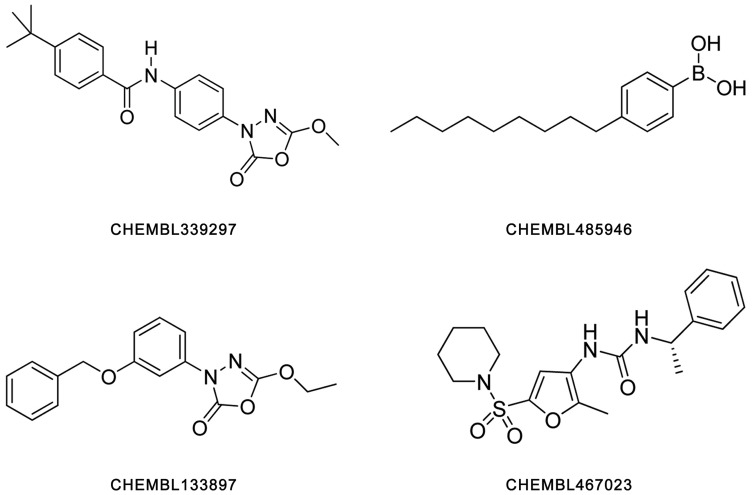
Constitutional formulae of four inhibitor compounds used for investigating binding poses with LPL, HL and EL.

All inhibitors used in docking study were retrieved from the ChEMBL database. Their biological values are described in Table 3, as reported previously [38]–[40]. For detecting the possible binding pockets of enzymes and investigating binding poses of small molecules, the top two inhibitors with the highest IC_50_ values for each lipase were selected (their constitutional formulae are shown in Figure 1).

**Table 3:** Inhibitors used in the docking investigation.

	CHEMBL ID of Inhibitor	IC_50_ (µM)	Binding Energy (kcal/mol)
**LPL Inhibitors**	CHEMBL339297*	0.20	−7.40
	CHEMBL485946*	1.40	−6.10
	CHEMBL1952294	20.00	−5.00
	CHEMBL1952314	500.00	−4.10
**HL Inhibitors**	CHEMBL339297*	1.80	−5.60
	CHEMBL133897	15.00	−5.90
	CHEMBL131588	>500.00	−3.70
**EL Inhibitors**	CHEMBL467023	0.06	−7.30
	CHEMBL485946*	0.10	−8.00
	CHEMBL1952299	22.00	−5.20
	CHEMBL1952301	50.00	−4.40

Table with their CHEMBL ID, experimental IC_50_ value, and predicted binding energy are listed.

* They are dual inhibitors.

## Results and Discussion

### Sequence Analyses and Template Selection

**Figure 2:**
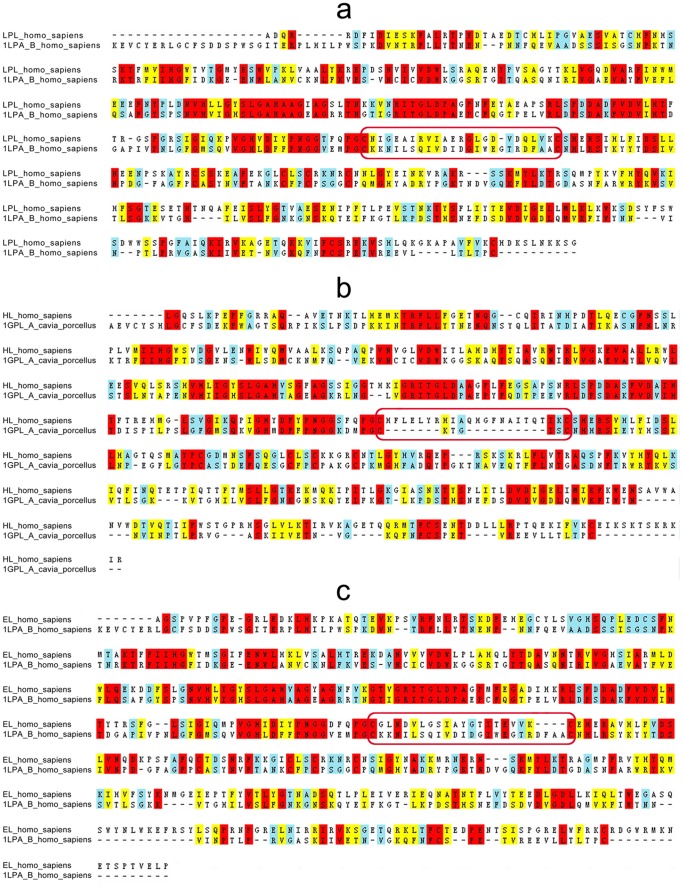
Sequence alignment between each lipase and their respective template. 1 LPA_B (Homo sapiens) and 1 GPL_A (Cavia porcellus) were used as templates for LPL (a), and EL (c) or HL (b). Red indicates identical amino acids, yellow indicates similar amino acids, and light blue designates somewhat similar amino acids. The amino acid sequences of lid elements of these lipases are marked with a red box.

**Figure 3:**
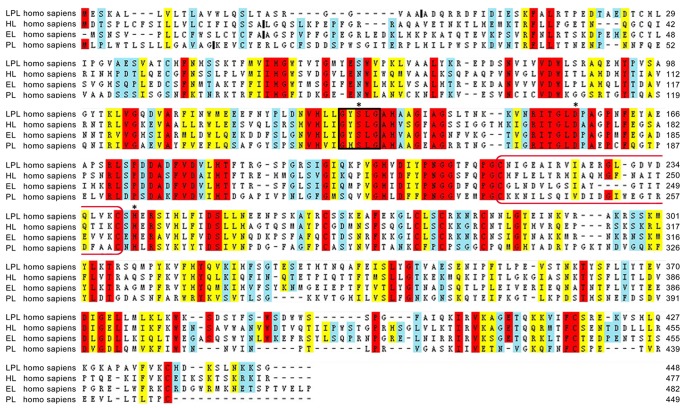
Multiple alignment of LPL, HL, and EL against the PL sequence. Red indicates identical amino acids, yellow indicates similar amino acids, and light blue designates somewhat similar amino acids. Amino acids are numbered according to convention, beginning with the first residue of the secreted protein. The predicted sites of signal peptide cleavage are marked with a solid line between amino acid residues. The GXSXG lipase motif containing the active serine is marked with black box. The amino acids of the catalytic triad are marked with an asterisk (Ser132, Asp156, and His241 in LPL, Ser146, Asp172, and His257 in HL, and Ser151, Asp175, and His256 in EL). The amino acid sequences of lid elements of the three lipases are marked with red boxes.

By aligning the sequences of three lipases against the sequences with known crystal structures, we found that Homo sapiens pancreatic triacylglycerol lipase (PDB entry: 1LPA with 3.04 Å resolution) [24] matches best with LPL and EL, and so was used as a template for homology modeling. In contrast, we found that the top two candidate templates for HL were pancreatic lipase-related protein 2 (PDB entry: 1 GPL with 2.01 Å resolution) [41] (ranked first), and 1 LPA (ranked second). We therefore used 1 GPL as a template for HL modeling (see below). There is 31%, 33%, and 35% sequence identity between the query sequences (LPL, HL, and EL, respectively) and their respective templates (1LPA_B and 1GPL_A) (Figure 2). 1 GPL is known to have a small lid element compared with HL and 1 LPA, so we further compared the sequences of the lid region (24 residues) of HL with 1 GPL and 1 LPA. We found that only three residues are identical between them (see the residues marked with red boxes in Figures 2b and 3). In subsequent homology modeling, the structure of the identical residues is automatically endowed from the template, while the coordinates of most non-identical residues are derived from the CHARMm residue topology library. The lid region of HL can therefore be conjectured. A random coordinate shift is attached or added to each atom in generated models to avoid too many similarities between the template and the target structure. Based on the sequence similarities (33% in 1 GPL *vs* 32% in 1 LPA), bit scores (192.2 in 1 GPL *vs* 191.8 in 1 LPA), and expectation values (E-values; 7.9e^−55^ in 1 GPL *vs* 1.5e^−54^ in 1LPA), we selected 1 GPL as the template for HL modeling definitively. The E-values for LPL, HL, and EL were 9.8e^−55^, 7.9e^−55^, and 1.7e^−55^, respectively. Because an E-value represents a number of different alignments with scores equivalent to, or better than, the scores that are expected to occur in a random database search, the low E-values of LPL, HL, and EL indicate that the alignments were real and did not occur by chance.

Additional multiple alignments were then performed between TLGS members and PL. The crystal structure of PL, a member of the human triacylglycerol lipase family with a closely genetic relationship to the subfamily containing LPL, HL, and EL, has been resolved [24]. The similarity between the three lipases and PL were 33.4%. In addition, all of them contain the classical “Ser-Asp-His” motif (Figure 3), which is consistent with previous studies [4]. The identified conserved characteristics and key residues were then used as the criteria for additional molecular dynamics exploration, binding pocket detection, and molecular docking studies.

### Generation, Refinement, and Evaluation of Homology Models

**Figure 4:**
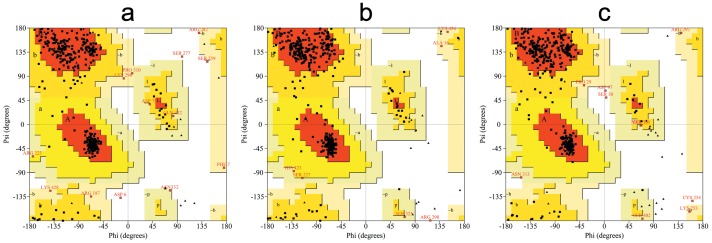
Ramachandran plot of the LPL, HL, and EL models. The different color codes indicate most favored (red), generously allowed (dark yellow), additionally allowed (light yellow), and disallowed (white) regions. For LPL, 84.5% of the residues were in the most favored regions, 12.4% in additionally allowed regions, 2.1% in generously allowed regions, and 1.0% in disallowed regions. In the case of HL, 84.7% of residues were in the most favored regions, 13.9% in additionally allowed regions, 1.2% in generously allowed regions, and 0.2% in disallowed regions. Similarly for EL, 87.3% of residues were in the most favored regions, 10.4% in additionally allowed regions, 1.5% in generously allowed regions, and 0.7% in disallowed regions.

Three-dimensional molecular models of the three lipases were generated using the B-chain of 1 LPA as the template for both LPL and EL, and using the A-chain of 1 GPL as the template for HL. The models constructed were stereo-chemically validated using additional parameters such as PROCHECK [25], and by analyzing residue-by-residue geometry and overall structural geometry (Figure 4). Most of the residues in these lipases were located in allowed regions (>98%), indicating that the quality of the models was reasonable and acceptable. This was confirmed by examining the models using profile-3D in DS 2.5 (see Table 1), and ProSA analysis (see Table 2).

It is well known that cofactors are required for enzymatic activation. ApoC-II is a key cofactor for LPL activation, while cofactors for HL and EL are yet to be defined [17]. We also considered whether the apoC-II-binding region might affect the interactions of inhibitors with LPL. It is known that the apoC-II binding region is located in different area, distant from the catalytic domain [17], which is a binding site for known inhibitors. It is therefore unlikely that apoC-II affects the interactions between inhibitors and LPL, based on the homology models generated in the current study. The models achieved were optimized using the molecular dynamics method described above. The final docking evaluation, as shown below, indicates that a simplified strategy for the designing and studying of TLGS inhibitors is reasonable. Nevertheless, it is necessary for future work to identify additional cofactors for HL and EL, and to investigate whether such cofactors will affect the interactions between inhibitors and the catalytic domains of each lipase.

**Figure 5:**
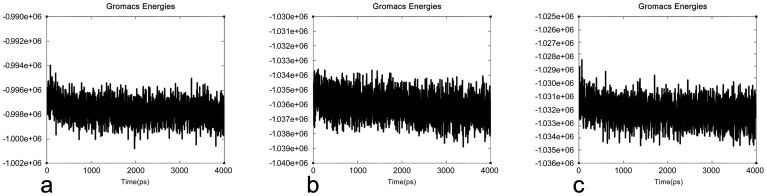
Potential energy plot of MDS. LPL, HL, and EL plots show the variation in potential energy throughout the system for a period of 4 ns. X-axis: time (ps). Y-axis: the potential energy (KJ/mol).

Initial models of TLGS were further refined using MDS to improve their stability. This strategy was also implemented to find energetically favorable structures for further docking analysis. The system of LPL contains one positive charge, and therefore one chloride ion was added to the model. For HL and EL, ten and four chloride ions were added to the models for MDS, respectively. The results, based on the trajectory, revealed that the potential energy of the LPL model decreased from −0.994 e+06 KJ/mol to −1.001 e+06 KJ/mol. However in the HL model, the decrease in potential energy varied from −1.034 e+06 KJ/mol to −1.039 e+06 KJ/mol. For the EL model, the decrease in potential energy varied from −1.028 e+06 KJ/mol to −1.035 e+06 KJ/mol. Most of the structures are in the region of 0.998 e+06 KJ/mol, 1.037 e+06 KJ/mol, and 1.032 e+06 KJ/mol for LPL, HL and EL, respectively. These results determine the energetic stability for LPL, HL and EL (Figure 5).

**Figure 6:**
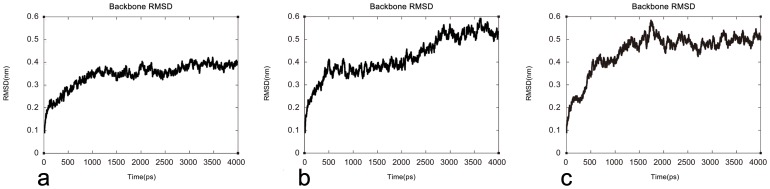
Graphical representation of the root mean square deviation (RMSD) plot. Backbone RMSD for LPL, HL, and EL from the initial structures throughout the simulation of 4 ns, as a function of time. X-axis: time (ps). Y-axis: RMSD (ns).

These models were further analyzed according to their structural stability using RMSD. One thousand ps were carried out for equilibrating. For LPL, a gradual rise was observed until 0.39 nm, and a plateau was observed thereafter. In the case of HL, a gradual rise was observed until 0.40 nm, before a plateau occurred for the rest of the study. Finally, there was a gradual rise until 0.49 nm, followed by a plateau, in the model for EL. The potential energy plots for LPL, HL, and EL are depicted in Figure 6. Profile-3D in DS 2.5 was then used to validate the models after MDS. During the ProSA analysis, the ProSA energy scores for the models were found to be better than those obtained for the models prior to MDS (Table 2). Overall, all the validation results above confirmed that the homology models constructed by our methods are satisfactory and reliable.

### Detection and Selection of Binding Pockets

**Figure 7:**
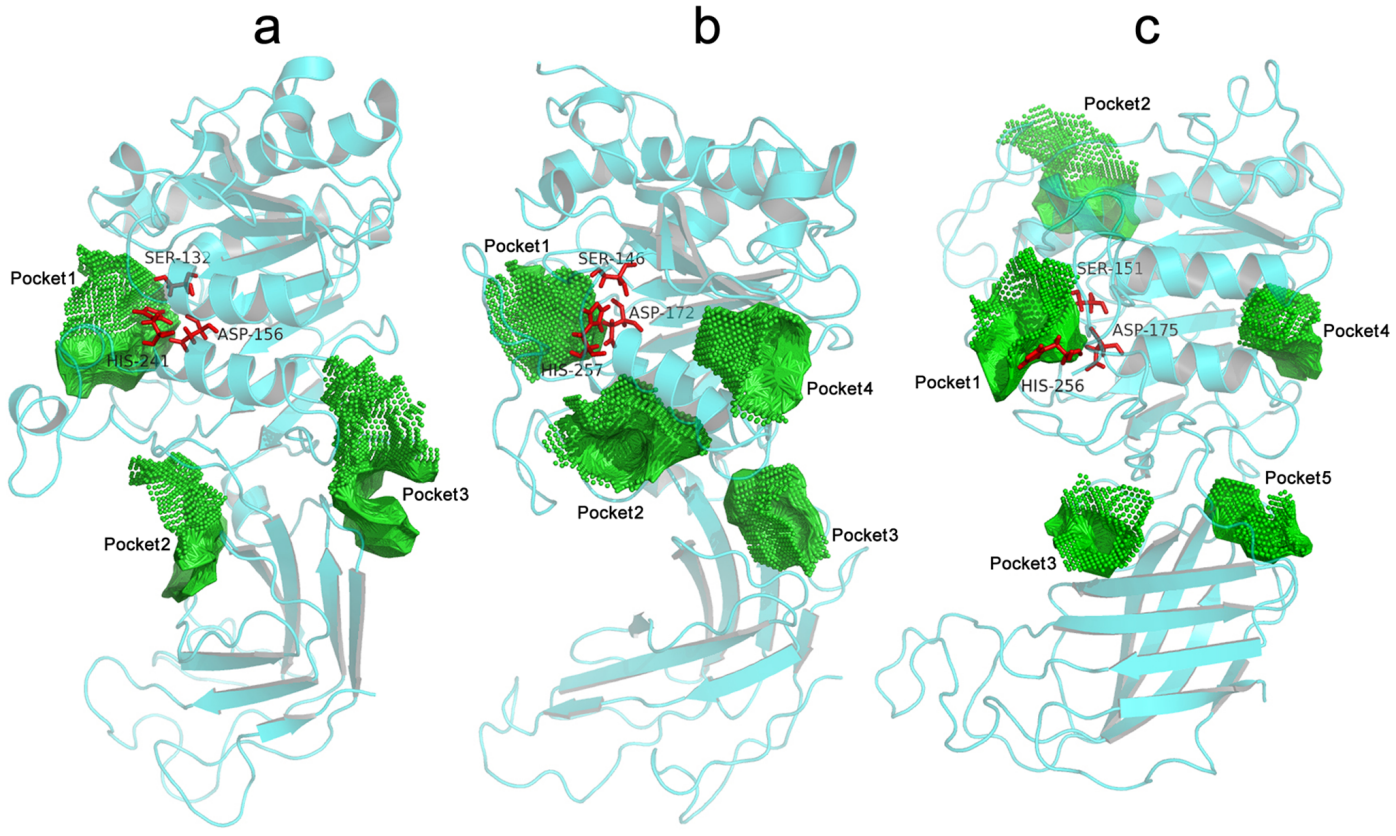
Views of putative binding pockets of LPL, HL, and EL. Predicted pockets of (a) LPL, (b) HL, and (c) EL are shown. The binding pocket information is created by Cavity. The key catalytic residues previously identified are all located in pocket 1. The graphics are generated using PyMOL program (http://www.pymol.org).

More than one possible ligand-binding pocket was proposed for each lipase by Cavity. In Figure 7, there are three potential binding pockets in LPL, and the best potential binding affinities (p*K*_d_) were predicted to be 10.47, 9.20, and 8.39. In the case of HL, four binding pockets were detected, and the potential best p*K*_d_ values were 9.28, 8.25, 7.92, and 7.41. For EL, a total of five binding pockets were suggested, and the potential p*K*_d_ values with ligands were 8.89, 8.76, 7.88, 7.87, and 6.76 for pockets 1–5, respectively. These possible binding pockets were then further filtered using the following two criteria: high predicted p*K*_d_ values (∼1 nM was used as the cut-off value for judging whether the binding pocket had the potential for achieving high binding affinity), and the location of the catalytic triad. The final proposed binding pocket is therefore pocket 1 of each TLGS member for the following structure-based investigations.

### Comparative Analysis and Structural Comparison of TLGS Members

In order to characterize the similarities and differences of the binding pockets of LPL, HL, and EL, the residues of three pockets proposed above (0.6 nm in diameter) were investigated.

**Figure 8:**
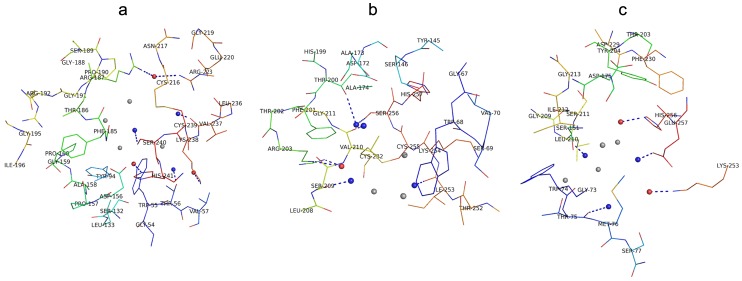
The pharmacophore features of (a) LPL, (b) HL, and (c) EL created by the Cavity approach. The red ball represents a “Hydrogen bond acceptor”, the blue ball represents a “Hydrogen bond donor”, and the gray ball represents a Van Der Waals and hydrophobic contact. Pharmacophore features located in binding pockets, and their corresponding residues, have been marked and labeled. The graphics are generated using the PyMOL program (http://www.pymol.org).

The residues of each lipase that may bind with ligands are shown in Figure 8, which includes some of our predicted residues based on previous site-directed mutagenesis studies [42]–[47]. We commonly identify point mutations in patients with triglyceride lipase deficiencies, and so our study provides reliable and significant models for further clinically relevant investigations.

**Figure 9:**
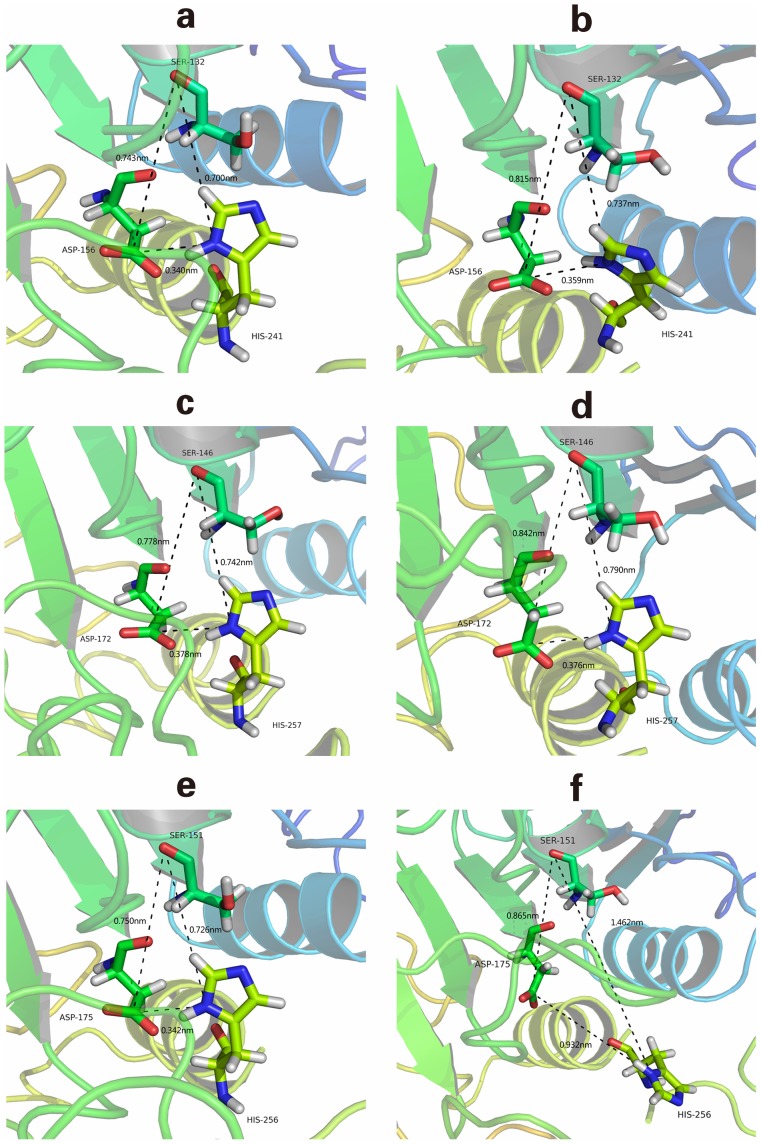
The triangles formed by the catalytic triad before and after MDS. From top to bottom on the left side: the structure and spatial triangle formed by catalytic triad residues before MDS. (a) LPL, (c) HL, and (e) EL. From top to bottom on the right side: the structure and spatial triangle formed by catalytic triad residues after MDS. (b) LPL, (d) HL, and (f) EL.

When considering the characteristics of the binding pocket, there were several issues that were always considered. One important consideration was whether spatial conservativeness could be determined for the conserved sequences and residues, which was important and meaningful for the realization of structure-based biological functions. Figure 9 shows the spatial positions and distances of the catalytic chemical groups located in the catalytic triad (a hydroxyl group in Ser, a carboxyl group in Asp, and the imidazole ring in His) before and after MDS. Before MDS, it was clear that there was an acute triangle formed by the catalytic triad of each TLGS member. The side lengths represented the distances between each chemical group, and the values were very similar, which may be a result of the sequence homology between TLGS members. After MDS, the spatial triangle of EL changed from an acute angle to an obtuse one, while the corresponding feature in LPL and HL retained the original shape and spatial positions. For EL, the distances between Ser151-His256 and Asp175-His256 were significantly increased. These results demonstrate that EL is somewhat flexible compared with LPL and HL. However, this flexibility was not strong enough to change the rigid structure of EL (Figures 5 and 6).

The orientation and spatial coordinates of the side chains included in binding pockets were further analyzed, which is important for structure-based interactions and molecular recognition. Generally, there are two main factors that can alter the orientation of a residues side chain. The first is distortion of protein backbone, and the second is the flexibility of the residue itself, both of which should be considered during the binding process. In Figure 9, the backbones in the binding pockets of LPL, HL, and EL are rigid. Although the side chains of the catalytic triad residues are somewhat flexible, the spatial orientation of these residues in both LPL and HL are stable, indicating that the binding pockets of LPL and HL are dynamically conservative. In EL, the spatial orientation of Ser151 and Asp175 were changed slightly, and His256 was obviously flipped, providing a direct reason why the spatial triangle changed from an acute to an obtuse angle during MDS.

Feature differences, especially in the shape of three lipase pockets, may be responsible for the differing IC_50_ values of their respective inhibitors (The subsequent docking studies reveal how differences in the pocket shape affect inhibitor selectivity and binding affinity). In Figure 8, there are three hydrogen bond acceptors (corresponding to Arg187 or Arg223, Lys238, and His241), and three hydrogen bond donors (corresponding to Thr56, Val237, and Ser240) in the LPL pocket. In the case of HL, one hydrogen bond acceptor (corresponding to Arg203) and up to four hydrogen bond donors (corresponding to Ser209, Val210, Thr200 or Ser256, and Ile253) were identified. For EL, there were two hydrogen bond acceptors (corresponding to Lys253 and His256), and three hydrogen bond donors (corresponding to Thr75, Leu210, and Glu257) in the binding pocket. The use of Arg residues (187 in LPL, and 203 in HL) as hydrogen bond donors is a feature shared by both LPL and HL. The use of Lys (238 in LPL, and 253 in EL) and His (241 in LPL, and 256 in EL) residues as hydrogen bond donors occurs in both LPL and EL. The hydrogen bond acceptor feature of using Thr (56 in LPL, and 75 in EL) occurs in both LPL and EL. These common features may therefore be the basis for binding of the dual inhibitors. Information regarding the binding mode and volume constraint differences of the three pockets could therefore be useful for designing selective inhibitors in the future.

### Docking Analyses

**Figure 10:**
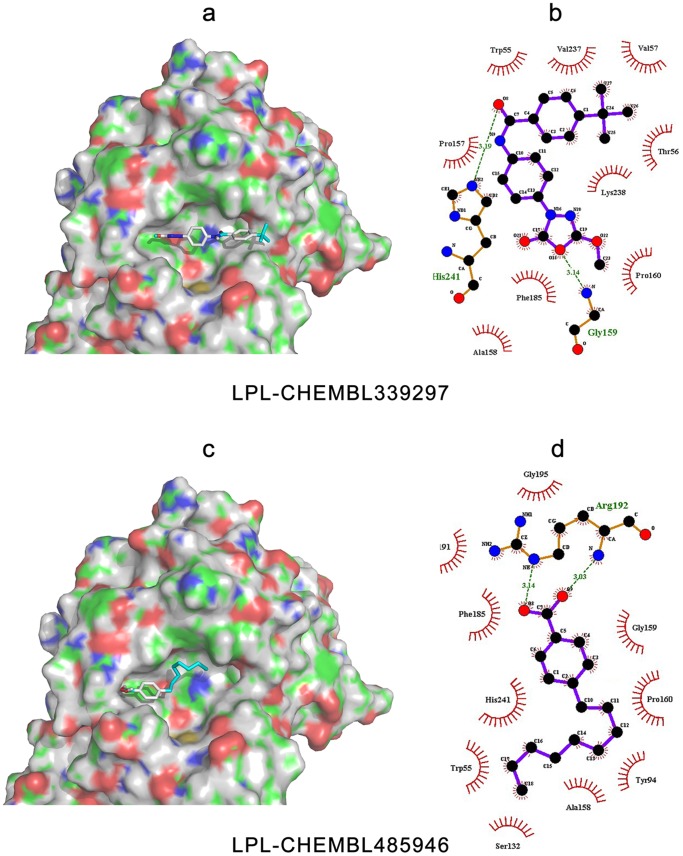
Binding modes and interactions of LPL with its inhibitors, CHEMBL339297 and CHEMBL485946. The pictures on the left side are of LPL complexed with (a) CHEMBL339297, and (c) CHEMBL485946. The protein surface and binding pocket is colored, with blue representing the positively charged region, red representing the negatively charged region, green representing the hydrophobic region, and gray representing the protein backbone. The pictures on the right side were created by LigPlot+ [48] for the representation of the interactions with (b) CHEMBL339297 and (d) CHEMBL485946, showing the inhibitors (purple), residues involved in hydrogen bonding with the ligand (brown), along with their hydrogen bonds (green), and residues involved in non-bonded interactions (red spikes).

**Figure 11:**
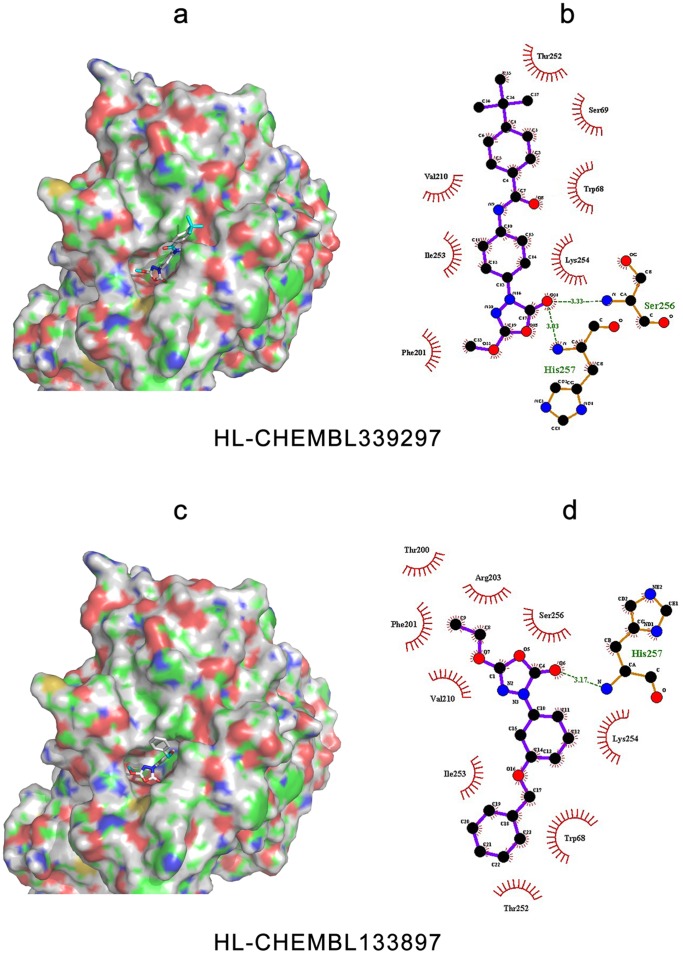
Binding modes and interactions of HL with its inhibitors, CHEMBL339297 and CHEMBL133897. The pictures on the left side are of HL complexed with (a) CHEMBL339297 and (c) CHEMBL133897. The protein surface and binding pocket is colored, with blue representing positively charged region, red representing the negatively charged region, green representing the hydrophobic region, and gray representing the protein backbone. The pictures on the right side were created by LigPlot+ to represent the interactions with (b) CHEMBL339297 and (d) CHEMBL133897, showing the inhibitors (purple), residues involved in hydrogen bonding with the ligand (brown), along with their hydrogen bonds (green), and the residues involved in non-bonded interactions (red spikes).

**Figure 12:**
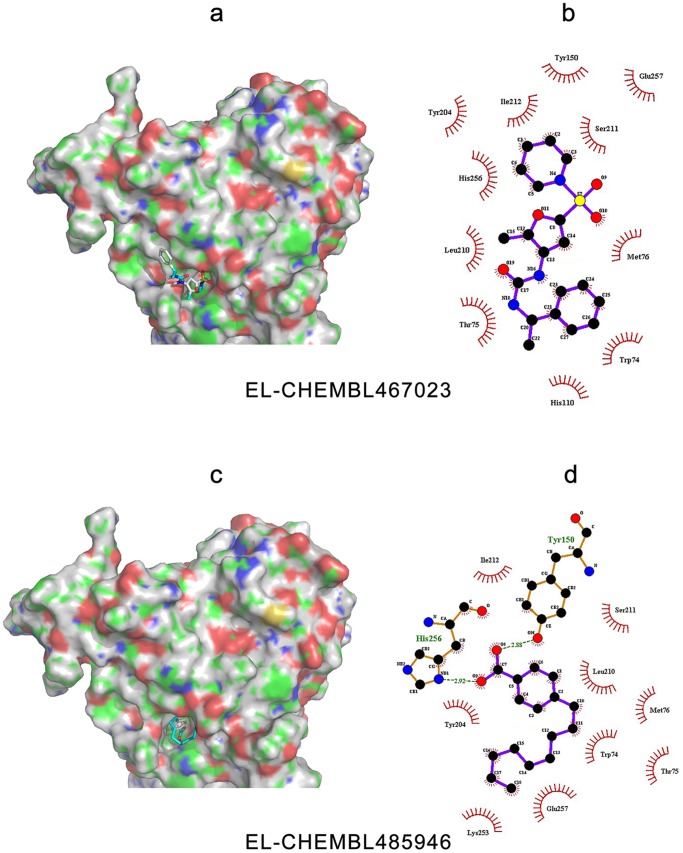
Binding modes and interactions of EL with its inhibitors, CHEMBL467023 and CHEMBL485946. The pictures on the left side are the EL complexed with (a) CHEMBL467023 and (c) CHEMBL485946. The protein surface and binding pocket is colored, with blue representing positively charged region, red representing the negatively charged region, green representing the hydrophobic region and gray representing the protein backbone. The pictures on the right side were created by LigPlot+ to represent the interactions with (b) CHEMBL467023 and (d) CHEMBL485946, showing the inhibitors (purple), residues involved in hydrogen bonding with the ligand (brown), along with their hydrogen bonds (green), and residues involved in non-bonded interactions (red spikes).

To further verify the feasibility of the homology models constructed above, and to investigate the spatial characteristics of the ligand binding properties, several inhibitors were selected for docking analysis. The protein-inhibitor interactions of each lipase with their two best inhibitors [38]–[40] are shown in Figures 10–12. Detailed analysis of each lipase and inhibitor are discussed below.

#### Prediction of binding patterns between potential inhibitors with LPL

When LPL accommodated its inhibitors, its binding pocket appeared open, shallow, and basket-like in shape (see Figure 10). Hydrophobic regions could be found surrounding the pocket, and some electrostatic features could also be found within it.

The two inhibitors were half-embedded into the binding site of LPL but did not completely fit within the pocket. This may help to explain why the IC_50_ values of LPL inhibitors were not good enough to reach nM degree. Compared with CHEMBL339297, CHEMBL485946 did not fit as well in to the pocket, and was localized to the corner of the binding site. The binding energies of CHEMBL339297 and CHEMBL485946 in the LPL pocket were −7.4 kcal/mol, and −6.1 kcal/mol, respectively, which explained why the experimentally calculated IC_50_ value of CHEMBL339297 (0.2 µM) was lower than that of CHEMBL485946 (1.4 µM).

The binding pose and pattern (Figures 10b and 10d) showed that CHEMBL339297 could form two hydrogen bonds with LPL (at His241 and Gly159). The His241 corresponded to a typical hydrogen bond acceptor, as described above. In contrast CHEMBL485946 formed a weak hydrogen bond with the carboxyl group of Arg192 in LPL, and the remaining interactions between the two molecules were mainly hydrophobic (including an aromatic stack effect between the benzene ring of the ligand, and residues Trp55, Tyr94, Pro160 and His 241 of LPL). This also explains why the IC_50_ of CHEMBL485946 was higher than that of CHEMBL339297.

Based on the binding poses and patterns, more work can be done to improve the biological activity of LPL inhibitors by increasing their molecular volume, the number of typical hydrogen bonds, and electrostatic interactions. In this way, it is possible to increase the binding capability of LPL inhibitors, and thus improve their effectiveness.

#### Prediction of binding patterns between potential inhibitors and HL

CHEMBL339297 and CHEMBL133897 were selected to investigate HL-inhibitor interactions [38]. CHEMBL133897 specifically inhibits HL activity, while CHEMBL339297 is a dual inhibitor for both LPL and HL.

The inhibitors were embedded in a narrow elongated pocket of HL (see Figure 11). Both ends of the pocket were electronegative, while the central region was mainly hydrophobic. The binding pocket of HL is obviously different from that of LPL, which is discussed in detail below. Since CHEMBL339297 is narrow and long in shape, similar to the binding site, it binds tightly within the binding pocket. In contrast, CHEMBL133897 only occupied a small region of the HL pocket, and the benzene ring was located outside the binding pocket. The binding differences of these two inhibitors may explain why the IC_50_ value of CHEMBL339297 (1.8 µM) is better than that of CHEMBL133897 (15 µM).

In addition, it is clear in Figure 11b and Figure 11d that CHEMBL339297 can form a single hydrogen bond with HL (either to Ser256, with a length of 0.333 nm, or with His257, with a length of 0.303 nm). Ser256 is corresponding to a typical hydrogen bond donor feature as mentioned above. CHEMBL133897, however, forms only a weak hydrogen bond with HL, with the oxygen atom of the carbonyl group bound to the nitrogen atom from the backbone of His257. This was not a predicted hydrogen bond donor as described above. There were also less hydrophobic contacts, which together explain why CHEMBL133897 is only a weak inhibitor of HL.

Based on the binding pattern of HL inhibitors in the pocket of HL, we can conclude that in order to increase the binding affinity of HL inhibitors, the first step is to consider how to introduce more electropositive groups at both ends to enhance the formation of additional hydrogen bonds and electrostatic interactions between the inhibitors and HL. The shape of the HL binding pocket should also be considered. Since the pocket is a dumbbell-like shape with a narrow linker space, and the narrowest part (0.36 nm) cannot be flattened down a heterocyclic ring, it may be beneficial to consider designing an inhibitor that is geometrically complementary with the linker space, to design suitable inhibitors that fit well within the HL binding site. Unfortunately, this will complicate the design process, and at the present time there are only five compounds identified as inhibitors of HL.

#### Prediction of the binding patterns between potential inhibitors and EL

Two types of structurally diverse small molecule inhibitors of EL have been reported in the literature [39], [40]. The most potent inhibitors, CHEMBL467023 (sulfonylfuran urea-type) and CHEMBL485946 (boronic acid-type) were selected here to investigate their interactions with EL. The EL binding pocket resembled a deep hole, and the main contributors to the interactions were hydrophobic contacts (see Figure 12). At the outside edge, the residues are somewhat electronegatively charged. The entire binding pocket comprises a shallow groove combined with the hole.

The cyclohexane region of CHEMBL467023, a specific inhibitor of EL, was deeply inserted in the EL pocket, and the rest of the inhibitor was located in the shallow groove. The structure of CHEMBL467023 is well matched with the EL binding pocket, and explains why its IC_50_ value was as good as 0.06 µM. The inhibitors could be further improved by altering the sulfanilamide and the benzene group in the shallow groove, and the linker arm between them. The compound CHEMBL485946 is a dual inhibitor that binds to both LPL and EL. Figure 12 reveals that the fatty acid chain of the benzene group plays a key role in its binding. When the length of the fatty acid chain was reduced [40], its inhibitory action is weakened, suggesting that the hydrophobic interactions between the inhibitor and lipase are important for the inhibitory activity.

Additional binding analyses are shown in Figures 12b and Figure 12d. CHEMBL467023 produced a very strong aromatic accumulation effect (using Trp74, Thr75, His110, Tyr150, Tyr 204, His256, and Glu257), and Van der Waals interactions (involving Met76, Leu210, Ser211 and Ile212). Although no typical hydrogen bonds or electrostatic interactions were identified, CHEMBL467023 was still able to bind to EL tightly, resulting in relatively strong inhibitory activity. CHEMBL485946 can form one hydrogen bond with Tyr150 or His256 (His256 retains typical pharmacophore features, as mentioned above). Because of its hydrophobicity, the molecular docking results show that CHEMBL485946 has a better affinity for EL than CHEBMBL467023, even though CHEMBL467023 is the more effective EL inhibitor. This suggests that the current docking algorithms may be defective for calculating atom-based contributions. Nevertheless, although the binding affinity of inhibitors may be positively correlated with their IC_50_ values, this correlation can be inconsistent because, in addition to binding affinity, the IC_50_ value can also be affected by other factors including the measurement methods, and the membrane permeability of the compounds.

#### Comparison of the binding pockets and poses of dual inhibitors

As stated above, both LPL inhibitors chosen were non-specific. In fact, when searching the CHEMBL database, we found that most LPL inhibitors could also inhibit EL, and consequently there are more inhibitors for EL than for LPL or HL. In the current study, the dual inhibitors CHEMBL339297 and CHEMBL485946, which are inhibitory for both LPL and EL, were further investigated.

The binding pockets of LPL and HL are quite different, as mentioned above (see Figure 10a and Figure 11a). Another HL inhibitor CHEMBL133897 is structurally similar to CHEMBL339297, suggesting that the chemical structure of the dual-inhibitor CHEMBL339297 may be stereo-selective towards HL. The binding pocket of LPL is large compared with the size of CHEMBL339297, meaning that their interactions are non-specific. Although CHEMBL339297 could bind to both LPL and HL, the binding patterns and poses were quite different. For LPL, CHEMBL339297 formed two hydrogen bonds with the lipase: one between the carbonyl group of ligand and the imidazole ring of His241, and the other between the oxygen atom of the cyclic ester, and the nitrogen atom of the backbone of Gly159. For HL, only one hydrogen bond was formed, and this was between the oxygen atom in the carbonyl group of CHEMBL339297, and the nitrogen atom from the backbone of Ser256 or His257. This may explain why CHEMBL339297 is more effective at inhibiting LPL than HL; even though it can bind to both lipases.

Although CHEMBL485946 is not considered drug-like by many medicinal chemists, its long flexible fatty acid chain means that it can bind to a large number of proteins. When binding to LPL and EL, the oxygen atom of its hydroxyl group forms a hydrogen bond with the lipases. When binding to LPL, a hydrogen bond is formed with Arg192, which is not predicted to be a typical pharmacophore feature above. In the case of EL, the boric acid group acts as an anchor, and allows CHEMBL485946 to bind deeply into the hole. The residue involved in hydrogen bond formation corresponded to His256, a typical pharmacophore as predicted. These results may explain why the inhibitory activity against EL is 10-fold stronger than against LPL.

#### Model validation with consistency between the predicted and experimental values

The docking studies described above using known inhibitors clarified the relationship between inhibitory activity and binding affinity, suggesting that the models of LPL, HL and EL constructed in the current study were reliable and useful. Importantly, a reliable protein model could be used not only to identify effective inhibitors, but also to distinguish the good from poor inhibitors. In order to evaluate the quality of these models, we selected inhibitors with different inhibitory activities [defined as follows: good (IC_50_<2 µM), moderate (2 µM ≤ IC_50_<50 µM) and poor (IC_50_≥50 µM)], and performed docking analysis with the constructed models of each lipase. Our results showed that there was consistency between the results of biological experiments and the predicted ΔG values (Table 3), indicating that the constructed models could effectively distinguish between good, moderate, and poor inhibitors. In particular, inhibitors with IC_50_>50 µM were considered to have no inhibitory activity (predicted pKi value less than 5), which was consistent with our docking scores. Taken together, these results consistently demonstrate that the constructed models of LPL, HL, and EL were reliable and accurate for the evaluation of inhibitor activity.

## Conclusions

In conclusion, we have systematically built homology models for all the TLGS members. In addition, we have identified the lipase binding pockets and residues of the lipases involved in binding, and also investigated the binding poses of specific and non-specific inhibitors towards TLGS members. Although LPL, HL, and EL belong to the same subfamily, their binding pockets are quite different. MDS indicated that the conformation of the EL active site changes more than LPL and HL during the catalytic process. This demonstrates that the EL pocket is more flexible when compared with the pockets of LPL and HL.

The lipase inhibitory activity is a result of blocking the catalytic site, rather than forming a strong interaction with the catalytic residues. Compound studies were therefore essential for designing effective lipase tools and inhibitors.

The proposed homology models could distinguish between good, moderate, and poor inhibitors. Consistent docking studies showed that the constructed models could be used to distinguish inhibitors that had potent inhibitory activity, suggesting that these models will be useful for designing efficient inhibitors and virtual screening in the future.

Our results provide important information on the molecular structures of lipases, indicating that the proposed models will be reliable for designing effective potential therapies for the treatment of hyperlipidemia and atherosclerosis.
